# Sigma Receptors as Endoplasmic Reticulum Stress “Gatekeepers” and their Modulators as Emerging New Weapons in the Fight Against Cancer

**DOI:** 10.3389/fphar.2018.00711

**Published:** 2018-07-10

**Authors:** Anna Tesei, Michela Cortesi, Alice Zamagni, Chiara Arienti, Sara Pignatta, Michele Zanoni, Mayra Paolillo, Daniela Curti, Marta Rui, Daniela Rossi, Simona Collina

**Affiliations:** ^1^Biosciences Laboratory, Istituto Scientifico Romagnolo per lo Studio e la Cura dei Tumori (IRCCS), Meldola, Italy; ^2^Pharmacology Section, Department of Drug Sciences, University of Pavia, Pavia, Italy; ^3^Laboratory of Cellular and Molecular Neuropharmacology, Department of Biology and Biotechnology ‘L. Spallanzani’, University of Pavia, Pavia, Italy; ^4^Medicinal Chemistry and Pharmaceutical Technology Section, Department of Drug Sciences, University of Pavia, Pavia, Italy

**Keywords:** sigma receptors, anticancer targeted therapies, chaperone activity, endoplasmic reticulum stress, cancer cell proliferation

## Abstract

Despite the interest aroused by sigma receptors (SRs) in the area of oncology, their role in tumor biology remains enigmatic. The predominant subcellular localization and main site of activity of SRs are the endoplasmic reticulum (ER). Current literature data, including recent findings on the sigma 2 receptor subtype (S2R) identity, suggest that SRs may play a role as ER stress gatekeepers. Although SR endogenous ligands are still unknown, a wide series of structurally unrelated compounds able to bind SRs have been identified. Currently, the identification of novel antiproliferative molecules acting via SR interaction is a challenging task for both academia and industry, as shown by the fact that novel anticancer drugs targeting SRs are in the preclinical-stage pipeline of pharmaceutical companies (i.e., Anavex Corp. and Accuronix). So far, no clinically available anticancer drugs targeting SRs are still available. The present review focuses literature advancements and provides a state-of-the-art overview of SRs, with emphasis on their involvement in cancer biology and on the role of SR modulators as anticancer agents. Findings from preclinical studies on novel anticancer drugs targeting SRs are presented in brief.

## Introduction

Over the past few decades, sigma receptors (SRs), including sigma 1 and sigma 2 receptor subtypes (S1R and S2R, respectively) have been widely associated with aging- and mitochondria-associated disorders, such as Parkinson’s and Alzheimer’s disease, multiple sclerosis and amyotrophic lateral sclerosis (Martin et al., 1976; Su, 1982; Vaupel, 1983; Quirion et al., 1987; Maurice and Lockhart, 1997; Skuza, 2003; Peviani et al., 2014; Collina et al., 2017b). Although no endogenous SR ligand has ever been found, progesterone has been identified as a potential candidate (Su, 1991; Monnet et al., 1995). This finding, together with a pressing need for new targeted therapeutic options for cancer, has led to important advances in what is known about the molecular structures and biological activities of SRs. However, the specific role played by this orphan receptor family in cell biology has yet to be clarified.

It was recently demonstrated that SRs are localized in plasmatic and subcellular membranes, in particular, the endoplasmic reticulum (ER) where they act as molecular chaperones stabilizing ER membrane proteins (Hayashi, 2015). The ER has a key role in the synthesis, folding, and structural maturation of more than a third of all the proteins produced in the cell, including almost all the secreted proteins (Anelli and Sitia, 2008). When misfolded proteins accumulate above a critical threshold as consequence of stressful conditions, a rapid and coordinated biochemical response involving adaptive signaling pathways [unfolded protein response (UPR)] is triggered (Schubert et al., 2000; Hetz et al., 2015). SR receptors can be considered as gatekeepers of ER stress, a condition that numerous studies have closely correlated with aging-associated diseases including cancer (Xu et al., 2005; Moenner et al., 2007; Brown and Naidoo, 2012; Schonthal, 2012; Yadav et al., 2014). We provide a state-of-the-art overview of S1R and S2R, focusing, in particular, on their involvement in cancer and on their potential role as ER stress gatekeepers. We also report on the compounds showing the greatest potential as biomarkers and effective drugs.

### S1R

**FIGURE 1:**
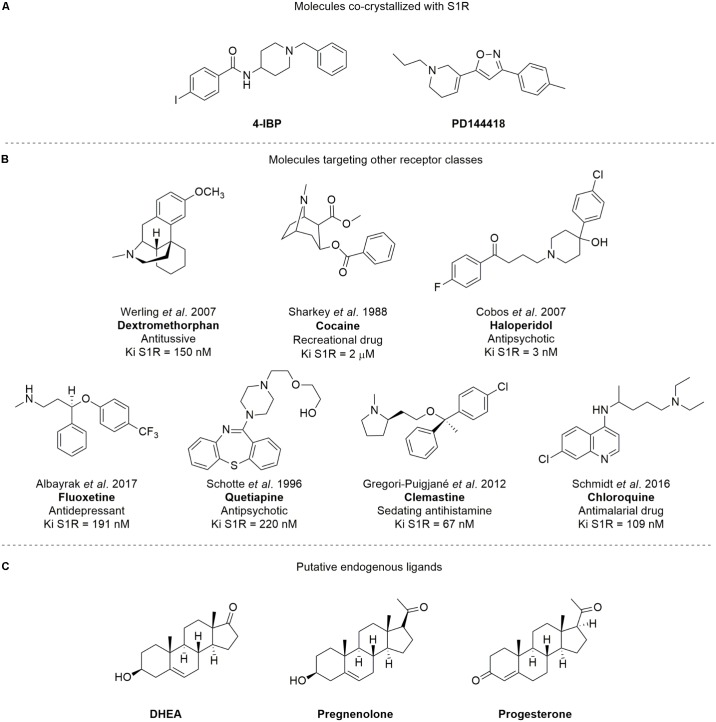
Structures of **(A)** compounds co-crystallized with S1R; **(B)** SR ligands targeting other receptor classes; **(C)** putative SR endogenous ligands. The Ki values showed are consistent with those previously reported from literature for Dextromethorphan (Werling et al., 2007), Cocaine (Sharkey et al., 1988), Haloperidol (Cobos et al., 2007), Fluoxetine (Albayrak and Hashimoto, 2017), Quetiapine (Schotte et al., 1996), Clemastine (Gregori-Puigjané et al., 2012), and Chloroquine (Schmidt et al., 2016), respectively.

S1R is an integral membrane protein of 26 kDa that is unrelated to any traditional transmembrane receptor (Quirion et al., 1992). Despite its small size, S1R is capable of modulating living systems, regulating the activity of numerous cellular proteins and is, in turn, modulated by a plethora of small molecules. The S*IGMAR1* (formerly *OPRS1*) gene was cloned in 1996, and its protein primary structure has long been known (Hanner et al., 1996). However, the overall three-dimensional structure and topology of its transmembrane architecture was unclear for a long time. Although, several potential structures of S1R have been postulated over the past decade (Hanner et al., 1996; Kekuda et al., 1996; Seth et al., 1998), the crystal structure of S1R [co-crystallized with 4-IBP and PD144418 (**Figure 1A**), PDB ID: 5HK1 and 5HK2, respectively] was only published in 2016 (Schmidt et al., 2016), revealing a trimeric architecture with a single transmembrane domain in each protomer. The carboxy-terminal domain of the receptor shows an extensive flat, hydrophobic membrane-proximal surface intimately associated with the cytosolic surface of the ER membrane. The domain includes a large, hydrophobic ligand-binding cavity at its center endowed with a remarkable plasticity in ligand recognition (Schmidt et al., 2016). This latter feature is in keeping with the most widely known function of S1R, i.e., a chaperone protein capable of interacting with several client proteins.

S1R has been detected at subcellular level primarily in the ER of various cell types in a range of tissues including the CNS, heart, ovaries, kidneys, testes, liver, pancreas, and placenta. High S1R expression has also been observed in embryonic stem cells and during the various stages of embryogenesis (Kekuda et al., 1996; Jbilo et al., 1997; Zamanillo et al., 2000; Ola et al., 2001; Langa et al., 2003; Aydar et al., 2004). In particular, S1R is mainly localized at the mitochondria-associated ER membrane (MAM), an interface between ER and mitochondria considered an important subcellular entity in that it acts as a sort of “tunnel” for lipid transport and Ca^2+^ signaling between these two organelles and contributes to processes required for cell survival (Hayashi and Su, 2007; Bernard-Marissal et al., 2015; Gregianin et al., 2016; Lewis et al., 2016; Watanabe et al., 2016). In MAM, S1R appears to play an important role as gatekeeper to keep ER stress under control. In brief, under conditions of stress, the level of Ca^2+^ decreases in the ER and S1R exits from a dormant state induced by its binding with the ER chaperone protein BiP (binding immunoglobulin protein), sustaining the proper conformation of the inositol triphosphate receptor type 3 (IP3), guaranteeing correct Ca^2+^ signaling from the ER to the mitochondria, and facilitating the synthesis of adenosine triphosphate (ATP; Hayashi and Su, 2007). Moreover, the ER is a unique milieu for the correct three-dimensional conformation of synthesized proteins, the level of which is maintained in a dynamic equilibrium between synthesis and degradation. Under conditions of stress, misfolded or aggregated proteins may accumulate within the ER, activating specific ER stress sensors, one of which is inositol-requiring enzyme 1 (IRE1). IRE1 is predominantly localized at the MAM interface where it is capable of detecting high levels of reactive oxygen species (ROS) produced by the mitochondria. Recent studies have shown that IRE1 is a client of S1R which, activated under ER and oxidative stress, chaperones IRE1, enhances its stability and guarantees the correct transmission of the ER stress signal to the nucleus, increasing the production of antistress and antioxidant proteins (Mori et al., 2013). In addition, S1R attenuates the formation of ROS by enhancing signaling of nuclear factor erythroid two-related factor 2 (Nrf2), a key regulator of antioxidant molecule expression (Wang et al., 2015).

Furthermore, S1R does not only reside in the ER but also in the plasma membrane, cytoplasmic membrane systems and nuclear envelope where it exerts a modulatory activity on different proteins. In particular, upon stimulation from agonists or stressors, S1R translocates to the plasma membrane to interact with ion channels, receptors, and kinases (Chu and Ruoho, 2016). It has also been shown to translocate to the nuclear membrane where it interacts with the nuclear envelope-resident protein emerin, recruiting a series of chromatin-remodeling factors that modulate gene transcription (Tsai et al., 2015).

Notably, S1R stands out from other chaperone proteins because of its unusual and promiscuous binding affinity to a wide series of molecules that target other receptor classes, such as dextromethorphan, cocaine, haloperidol, fluoxetine, quetiapine, clemastine, and chloroquine (Su et al., 2010; **Figure 1B**). It has been suggested that neurosteroids [e.g., dehydroepiandrosterone (DHEA), pregnenolone, and progesterone; **Figure 1C**] may be S1R putative endogenous ligands, despite their low binding affinities (0.3–10 μM; Su et al., 1988; Bergeron et al., 1996; Urani et al., 2001).

Overall, these observations point toward S1R being a new class of macromolecules halfway between a chaperone protein and a co-activator of receptors, which are activated by cell machinery to survive under conditions of stress. In support of this, there is growing evidence that S1R is only active in conditions of stress, remaining ‘silent’ in healthy organs (Maurice and Su, 2009; Tsai et al., 2009).

**FIGURE 2:**
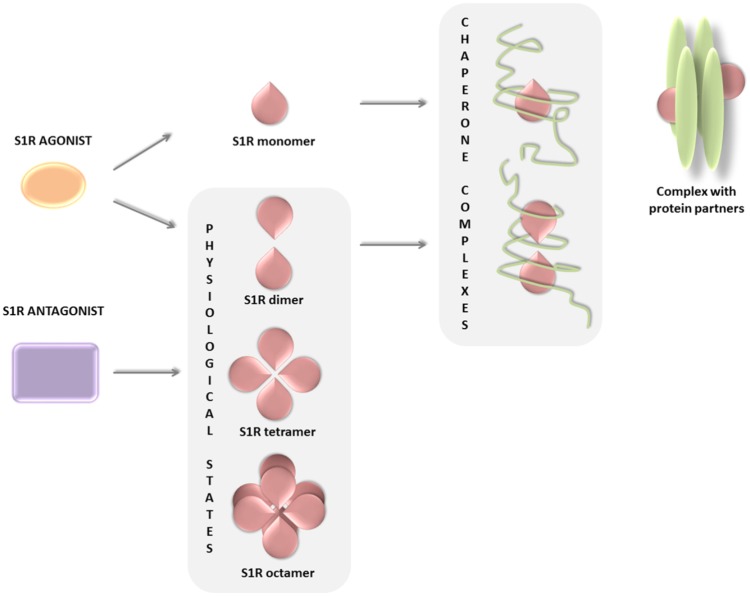
S1R ligands and their ability to change the oligomerization status of the receptor.

The biological response following ligand binding remains only partially understood and appears to be related to the oligomerization properties of S1R. The mechanistic models proposed (Mishra et al., 2015; Chu and Ruoho, 2016) suggest that the receptor changes its oligomerization status after binding with its ligands, some stabilizing the formation or the stabilization of S1R monomeric, dimeric, and higher oligomeric complexes. Thus, dimer and monomer forms may be functional chaperone states, whereas higher oligomeric complexes of S1R may act as a repository for the active forms. In addition, the S1R monomer is known to bind to protein partners on the plasma membrane, forming a functional unit potentially indicative of a secondary function of S1R and independent of its chaperone activity (**Figure 2**; Gromek et al., 2014; Mavlyutov et al., 2015; Bolshakova et al., 2016). In conclusion, the equilibrium of S1R in different states of oligomerization, i.e., monomers, dimers, or higher oligomeric forms, may explain its multiple interactions with such a wide number of heterogeneous classes of proteins.

### S2R

Over the years, pharmacological, chemical, and biological papers have demonstrated that S2R is a potential therapeutic target for several diseases including neurodegenerative disorders and cancer (Vilner and Bowen, 1993; Vilner et al., 1995; Bowen, 2000; Wheeler et al., 2000; Crawford and Bowen, 2002; Kashiwagi et al., 2009; Hornick et al., 2010; Guo and Zhen, 2015). Based on indirect evidence of S2R overexpression in peripheral and cerebral tumors, it has been hypothesized as a potential target for anticancer therapy (Crawford and Bowen, 2002; Crawford et al., 2002; Rui et al., 2016), and S2R radiotracers have been developed to image tumors (Tu et al., 2005, 2007, 2010; Mach and Wheeler, 2009; Mach et al., 2009). However, despite the numerous studies performed to date in this setting, the unknown molecular identity of the receptor has limited biological investigations and hindered the search for new drugs that act via the S2R pathway.

Xu et al. (2013) hypothesized that S2R is a part of the progesterone receptor membrane component 1 (PGRMC1) complex and since numerous articles have been published based on this supposition (Yang et al., 2002; Suchanek et al., 2005; Peluso et al., 2006, 2010; Cahill, 2007; Rohe et al., 2009; Intlekofer and Petersen, 2011; Szczesna-Skorupa and Kemper, 2011; Ahmed et al., 2012; Mir et al., 2012, 2013; Bali et al., 2013; Izzo et al., 2014a,b). However, the hypothesis, albeit appealing, had some serious weaknesses including the discrepancy between the molecular weight of PGRMC1 and S2R, and the low-binding affinity of PGRMC1 for haloperidol, the latter considered a characteristic signature of S2R (Hellewell and Bowen, 1990; Walker et al., 1990; Pal et al., 2007; Abate et al., 2015; Chu et al., 2015; Van Waarde et al., 2015).

In a recent paper, Alon et al. (2017) purified the putative S2R from calf liver tissue and attributed its identity to TMEM97, a relatively unknown protein belonging to the TMEM (transmembrane) gene family, resident in ER, involved in cholesterol homeostasis (Bartz et al., 2009) and in Niemann–Pick type C disease as NPC1-interacting protein (Ebrahimi-Fakhari et al., 2015). Cellular cholesterol homeostasis is a process of central importance and highly regulated. Dysregulation of the biosynthesis and uptake of cholesterol and cellular lipid accumulation has been correlated with ER stress and activation of the UPR (Colgan et al., 2011). The authors showed that the pharmacologic profile of TMEM97 is the same as that of S2R and that TMEM97 ligands bind S2R (Alon et al., 2017). The 3D structure of TMEM97/S2R, once understood, could shed fundamental light on its biological functions and their potential involvement in a broad spectrum of driver pathways of cancer and neurodegenerative diseases, thus facilitating the development of novel effective drugs.

## Er Stress and Srs

Current literature data, including recent findings on S2R, suggest that SRs are not a receptor family, a hypothesis further supported by the lack of endogenous ligands and by the SR capability to bind different proteins. Some experimental evidences suggest the pivotal role of SRs in ER stress response. First, SRs are predominantly expressed in ER and over expressed in several pathological conditions (i.e., cancer and neurodegenerative diseases). Furthermore, the S2R identity with TMEM97, a transmembrane protein of ER involved in cholesterol homeostasis has been demonstrated. Under physiological conditions, chaperones resident in the cytosol and ER lumen ensure precise folding of newly synthesized native proteins. ER stress due to accumulating misfolded proteins triggers a signaling reaction referred to as UPR (Hetz et al., 2015) that is committed to restoring ER protein homeostasis (or proteostasis) by increasing protein-folding capacity to ensure cell survival and normal functioning (Walter and Ron, 2011). However, in the event that UPR fails to restore a physiological protein equilibrium, the same ER sensors trigger an alternative response known as “terminal UPR,” leading to cell death (Shore et al., 2011; Oakes and Papa, 2015).

There is now evidence that ER stress is a driver of physiological and pathological brain aging (proteinopathies or protein misfolding disorders) and that neuronal UPR influences global proteostasis at the whole organism level (Martínez et al., 2017). Furthermore, numerous authors have demonstrated a high activation of the UPR machinery in several human solid tumors, including glioblastoma and breast, stomach, esophageal, and liver cancer (Fernandez et al., 2000; Shuda et al., 2003; Moenner et al., 2007). This is hardly surprising as cancer cells often spread to unfavorable environments characterized by hypoxia, low pH, high levels of ROS and inadequate glucose and amino acid supply, all of which may compromise ER protein folding (Ma and Hendershot, 2004; Koumenis, 2006; Lee and Hendershot, 2006; Moenner et al., 2007). Moreover, intrinsic stresses common to many tumor cells due to their genomic instability may lead to increased protein synthesis and secretory activity (Tollefsbol and Cohen, 1990; Ruggero, 2013; Dejeans et al., 2014; Horne et al., 2014).

**FIGURE 3:**
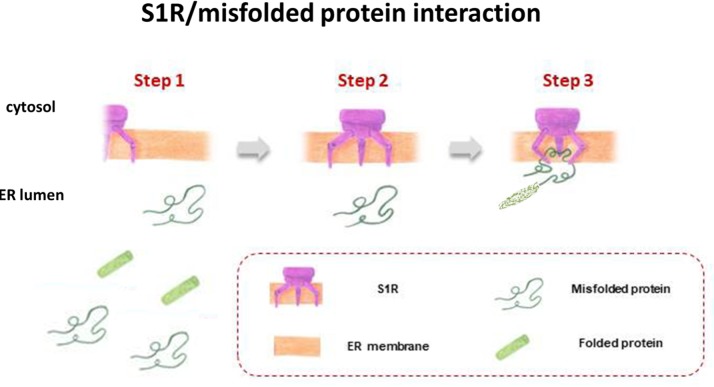
S1R acts as a chaperone protein to restore the correct folding of misfolded proteins.

**FIGURE 4:**
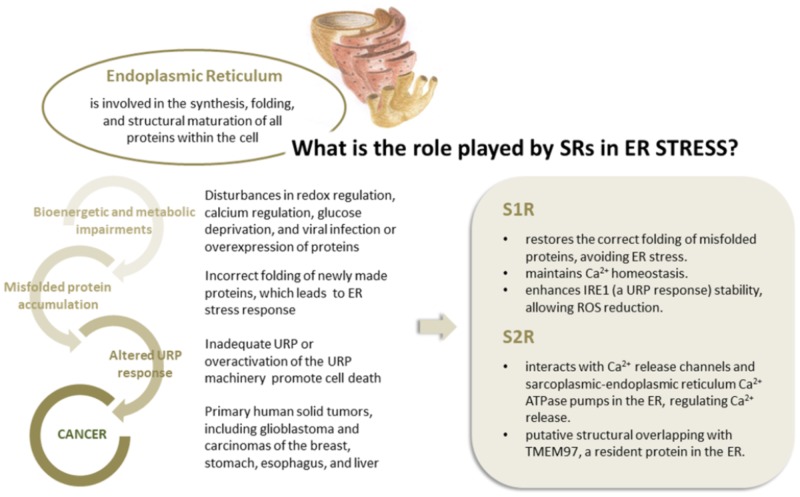
The correlation between ER stress and cancer conditions. The role played by S1R and S2R in modulating ER stress.

S1R, integrated into UPR machinery, may act as a chaperone protein to restore the correct folding of misfolded proteins (mainly ion channels, but also transcription factors and kinases), providing an escape route for chronically damaged cells that would otherwise die in response to ER stress (**Figure 3**). The same may be true for S2R/TMEM97, the biological role of which has yet to be clarified, but indirect evidence points toward its being one of the key factors in ER stress management. This may be because S2R belongs to the TMEM protein superfamily, a group of about 310 different proteins considered constituents of cell membranes such as ER, mitochondrial membranes, and lysosomal and Golgi apparatus. The function of the majority of TMEM proteins has yet to be clarified, principally because of problems in extracting and purifying transmembrane proteins (the same difficulty encountered in the unveiling of the molecular identity of S2R; Gebreselassie and Bowen, 2004; Palmer et al., 2007). However, some of these proteins are thought to be involved in conditions of ER stress, e.g., transmembranous anion channels (ANO1; Fuller, 2012), molecules responsible for oncosis (TMEM123; Ma et al., 2001), protein glycosylation (TMEM165; Foulquier et al., 2012), pathogen intoxication (TMEM181; Carette et al., 2009), and innate immunity response (TMEM173; Ishikawa and Barber, 2008). This last protein is currently arousing great interest in the area of cancer research (Harding, 2017). In addition, the S2R/TMEM97 may exert antiproliferative effects in actively proliferating tumor cells, attenuating ER stress. This assumption is probably correlated to the ability of TMEM97 in modulating cholesterol homeostasis, since a deregulation of this sophisticated mechanism leads to the activation of UPR machinery to restore the physiological conditions (**Figure 4**; Colgan et al., 2011).

Further substantial evidence of the close relation between S2R and ER activity is the ability of S2R ligands to influence the release of Ca^2+^ from this organelle. Although the mechanism behind this behavior is not fully understood, S2R seems to interact, directly and indirectly, with the Ca^2+^ release channels-IP3-gated Ca21 channel (IP3 receptor), ryanodine-gated Ca^2+^ channel (ryanodine receptors), and the sarcoplasmic-ER Ca^2+^ ATPase (SERCA) pumps in the ER, regulating the release of Ca^2+^ (Vilner and Bowen, 2000; Mach et al., 2013; **Figure 4**).

### SRs and Cancer

Several studies suggest that the deregulation of S1R may be involved in several human diseases, including cancer. In fact, S1R overexpression is associated with an invasive and metastatic phenotype in many human tumors, whereas low expression levels are found in normal cells (Bem et al., 1991; Spruce et al., 2004; Wang et al., 2004; Aydar et al., 2006; Skrzycki and Czeczot, 2013; Xu et al., 2014; Gueguinou et al., 2017; Kim and Maher, 2017). In the last decade, the correlation between S1R and cancer cells has been extensively studied, leading to hypothesize its functions in tumor biology and to investigate its therapeutic implications in cancer (see, as example, Achison et al., 2007; Palmer et al., 2007; Crottés et al., 2013; Kim and Maher, 2017). In response to environmental conditions occurring in tumor tissue, S1R may activate different adaptation mechanisms on the basis of the client protein present in a given cancer cell type (Crottés et al., 2013).

Moreover, radioligand binding assays highlighted a high density of S1R in neuronal and non-neuronal tumors (i.e., surgical specimens of renal and colorectal carcinoma and sarcoma), leading to the hypothesis of an important role of S1R in cancers (Kim and Maher, 2017).

Of note, S1R modulates the activity of several ion channels, promoting cell proliferation and survival. Several studies indeed suggested that ion channels constitute one of the main client protein families for S1R (Carnally et al., 2010; Crottès et al., 2011; Balasuriya et al., 2012; Kourrich et al., 2012). Notably, ion channels have long been considered involved in key aspects of cancer progression, including mitosis, migration, apoptosis, adhesion to the extracellular matrix (ECM) angiogenesis, homing, and drug resistance (Lang et al., 2004; Weber et al., 2006; Pillozzi et al., 2007; Gillet et al., 2009; Becchetti and Arcangeli, 2010; Pillozzi and Arcangeli, 2010; Becchetti, 2011). This may explain why cancer cells are capable of adapting to adverse metabolic conditions present in tumor tissue (Wulff and Castle, 2009; Prevarskaya et al., 2010; Arcangeli, 2011). Although the modulation of ion channel expression in cancer cells is still not fully understood, it is thought to be a consequence of the acquisition of an undifferentiated phenotype. Indeed, it is acknowledged that tumors, unlike healthy tissue, often show high levels of ion channels and transporters (Peruzzo et al., 2016).

**FIGURE 5:**
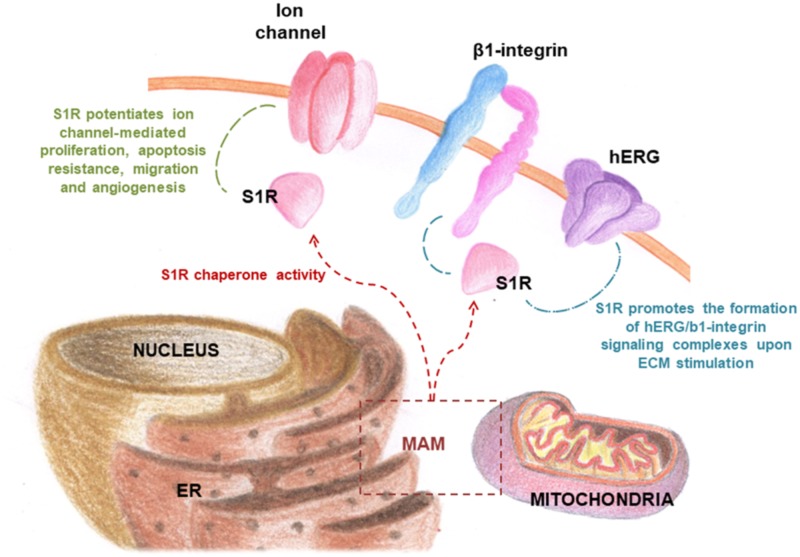
S1R chaperone modulates the activity of numerous ion channels in cancer cells.

In this respect, it has been hypothesized that S1R may be involved in the remodeling of cancer cell electrical properties, potentiating ion channel function associated with proliferation, cell death resistance, invasion, and angiogenesis (**Figure 4**; Crottés et al., 2013). Recently, it was shown that S1R influences cancer cell behavior by modulating membrane electrical characteristics in response to the ECM properties and stimuli (Crottès et al., 2011), thus activating the PI3K/AKT pathway, cell motility, and VEGF secretion (**Figure 5**). *In vivo*, S1R expression increased tumor aggressiveness by enhancing invasion and angiogenesis, and reducing survival (Crottés et al., 2016).

In addition, studies analyzing the promoter region of the S1R gene highlighted the presence of a number of binding sites for several transcription factors. Some are frequently involved in cancer cell proliferation, including two nuclear factor NF-κB, activator proteins (AP-1 and AP-2), globin transcription factor 1 (GATA-1), interleukin six responsive element (IL6RE), and steroid-response elements (Prasad et al., 1998). Of note, it was shown that the use of S1R antagonists prevented nuclear translocation of androgen receptor (AR), induced proteasomal degradation of AR and its splice variant, ARV7 (frequently detected in castrate-resistant prostate cancer), and consequently suppressed their transcriptional activity (Thomas et al., 2017). Within this context, S1R probably acts as a chaperone or scaffolding protein that coordinates the maturation and transport of client proteins crucial for physiological AR function.

**FIGURE 6:**
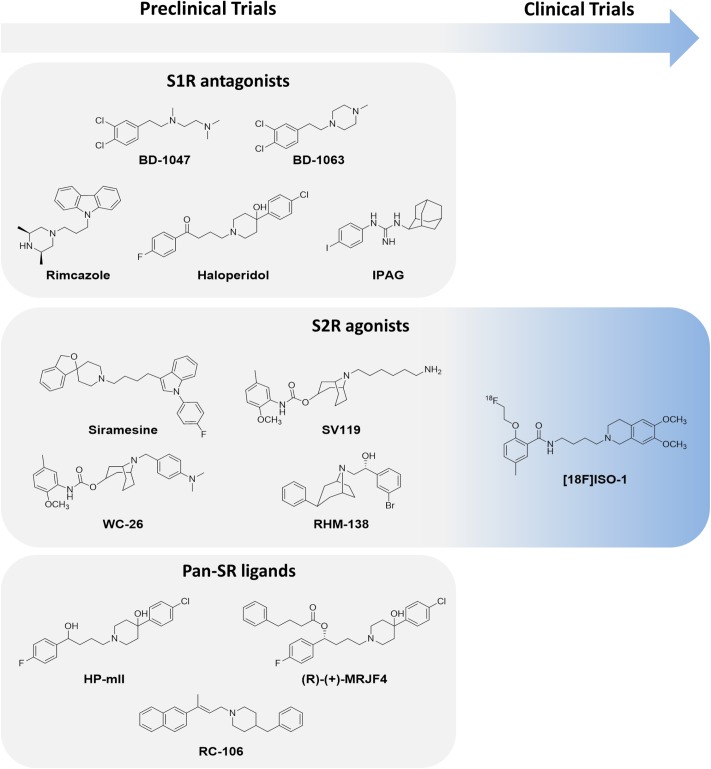
S1R and/or S2R modulators in preclinical or clinical trials.

Like S1R, S2R is also highly expressed in proliferating tumor cells, whereas low expression is observed in normal quiescent cells (Mach et al., 1997; Wheeler et al., 2000; Colabufo et al., 2006). Data currently available on S2R mechanisms of action derive from pharmacological experiments aimed at evaluating the impact of S2R-selective ligands on tumor cell biology. Although findings indicate that S2R plays a pivotal role in regulating tumor cell proliferation, survival, and invasion, its mechanisms of action and biochemical role in intracellular signaling pathways are still unclear. Within this context, several experiments have been performed to elucidate the molecular cascades behind S2R activation, including caspase-mediated apoptosis, autophagy, and cell cycle impairment (Zeng et al., 2012). Over the years, numerous studies have been performed to unambiguously define the mechanism of action linked to S2R. Česen et al. (2013) showed that siramesine (**Figure 6**), the gold standard of S2R agonists (Ostenfeld et al., 2005; Groth-Pedersen et al., 2007; Rui et al., 2016), triggers cancer cell death through mitochondria destabilization. In brief, siramesine induces ROS generation, which leads to the peroxidation of cardiolipin and the release of cytochrome *C* from the mitochondria. Thus, the effect of siramesine on mitochondrial membranes may functionally disable the mitochondria and alter cell homeostasis, thereby initiating cell death. Another study reported on the ability of S2R agonists to regulate the sphingolipid metabolic cascade. Sphingolipids are essential molecules in the process of cell proliferation and differentiation, and impairment of their biosynthetic pathway may lead to apoptosis and cellular motility (Crawford et al., 2002). A p53- and caspase-independent apoptotic pathway differing from that activated by alkylating, antiblastic drugs has also been ascribed to S2R selective agonists. This distinctive cytotoxic effect of S2R agonists could thus be useful to treat metastatic cancer (Crawford and Bowen, 2002). Moreover, Hornick et al. (2010) found that cell death can be induced by S2R agonists through early permeabilization of the lysosomal membrane and protease translocation, which trigger downstream effectors of apoptosis. Conversely, other authors observed that some S2R ligands are capable of mobilizing intracellular calcium ions and modulating potassium channels (Vilner and Bowen, 2000; Cassano et al., 2006, 2009), leading to an incorrect ionic balance and ultimately to cell death. All of these studies showed that the multiple pathways triggered by S2R are dependent on both the selected S2R ligand and the cell line under evaluation. Summing up, both S1R and S2R seem to be involved in cancer progression.

### SR Modulators and Cancer

Interest in SRs has increased substantially over the years, as documented by the high number of research papers and reviews published, patents registered (Bowen, 2000; Bourrie et al., 2004; Collina et al., 2013, 2017a; Georgiadis et al., 2017), as well as by the presence of SR modulators in the pipelines of pharmaceutical industries^1^^,^^2^^,^^3^ (Accuronix Therapeutics, 2018; Anavex Pipeline, 2018; Context Therapeutics, 2018).

Modulators of SRs have historically been categorized as antagonists or agonists basing on their ability to activate or inactivate the receptors and therefore on their pharmacological behavior (Martin et al., 1976; Mei and Pasternak, 2002; Mégalizzi et al., 2007; Su et al., 2010; Nguyen et al., 2015).

Regarding S1R subtype, it has been evidenced that S1R antagonists are able to prevent the activation of specific S1R-ion channel pathways; this represents a straightforward strategy against tumors (Crottés et al., 2013). Within this context, numerous anticancer molecules endowed with S1R antagonist behavior have been introduced into the pharmacological arena. BD-1047 and BD-1063 (**Figure 6**) are two such molecules that emerged by virtue of their ability to reduce proliferation in breast cancer cell lines (Hajipour et al., 2010). However, despite promising preclinical profiles, neither has reached the clinical experimentation phase.

Recent studies suggest that a valid alternative to the development of novel drug candidates against S1R could be the re-evaluation of well-established S1R modulators such as rimcazole, haloperidol, and IPAG (1-(4-Iodophenyl)-3-(2-adamantyl)guanidine; Schrock et al., 2013; **Figure 6**). These compounds can be defined as inducers of apoptosis involving NF-κB pathway in lung, Hodgkin’s lymphoma, and breast cancer cell lines (Spruce et al., 2000) or regulating ER stress, ROS production and translational repression (Kim et al., 2012; Happy et al., 2015). Moreover, they promote antiproliferative and antiangiogenic mechanisms in breast carcinoma xenograft (Spruce et al., 2001; Gilmore et al., 2004). Of note, it was recently suggested that S1R is involved in antitumor immunity mediated by the PD-1/PD-L1 checkpoint pathway, a known mechanism that allows tumor cells to escape immune surveillance and circumvent the generation of an immune response against the tumor. Maher et al. (2018) provided evidence of the ability of S1R to co-localize and physically bind to PD-L1, also demonstrating that the pharmacologic inhibition of S1R decreases PD-L1 cell surface expression and immune checkpoint activity in *in vitro* models. In particular, the authors reported that the exposure of cancer lines to the S1R modulator IPAG (1-(4-Jodophenyl)-3-(2-adamantyl) guanidine) induced selective autophagic degradation of PD-L1. This suggests that S1R modulators could be potential therapeutic agents in strategies aimed at inducing an immune response against cancer cells. In conclusion, a growing body of evidence points toward the potential of S1R ligands as anticancer therapeutic agents. However, the multiple roles of this protein in cancer biology need to be better clarified through further research.

There is also evidence that S2R modulators may be promising drugs against cancer, even if some functional and structural aspects of SR2 have yet to be elucidated (Wheeler et al., 2000; Zeng et al., 2012). Moreover, the putative overlapping of the pharmacological activity and ligand binding profile of S2R with TMEM97, a protein overexpressed in some tumor types, reinforces the idea that this SR subtype could be a marker for tumorigenesis (Alon et al., 2017). The definition of the crystal structure of TMEM97/S2R, as yet unknown, will be crucial in designing new chemical entities with a high affinity for this receptor. So far, effective S2R agonists have been discovered using a ligand-based approach (Ostenfeld et al., 2005; Groth-Pedersen et al., 2007; Chu et al., 2009; Tu et al., 2010; Petersen et al., 2013; Rhoades et al., 2013). Of note, some molecules belonging to different chemical classes have been extensively investigated in experimental studies. Recent research carried out on mouse breast cancer, human, or murine pancreatic cancer and human melanoma cell lines has shown that some S2R agonists, i.e., siramesine, SV119, WC-26, and RHM-138 (**Figure 6**), exert a cytotoxic effect at very low concentrations (Kashiwagi et al., 2007; Zeng et al., 2012). However, although S2R agonists are promising pharmaceutical/therapeutic tools, there is still a long way to go before they can be implemented into clinical practice. Only one compound, [18F]ISO-1, a potential PET marker of cell proliferation, is currently being evaluated in a phase I clinical trial (Dehdashti et al., 2013).

An innovative strategy that has also been successfully applied to the area of SRs and could potentially constitute an effective treatment for cancer is the use of dual target molecules. Given the high potential of this therapeutic approach, research has been focused on identifying compounds endowed with an S1R antagonist/S2R agonist profile, defined by Rui and colleagues as “pan-SR ligands” (Rui et al., 2016; Rossi et al., 2017). Marrazzo, the forefather of pan-SR therapy, showed that a haloperidol metabolite HP-mII (**Figure 6**) was effective against both SR subtypes and induced a modest antiproliferative activity in LNCaP and PC3 prostate cancer cells and in rat C6 glioma cells (Kashiwagi et al., 2007; Marrazzo et al., 2011). Recently, HP-mII was totally synthesized and functionalized with 4-phenylbutanoyl chloride, accessing the prodrug (*R*)-(+)-MRJF4 (**Figure 6**). This molecule possesses a more pronounced ability to reach the CNS and induce the death of rat C6 glioma cells than the original HP-mII molecule (Sozio et al., 2015). RC-106 (**Figure 6**), belonging to the pan-SR category, was recently evaluated in a panel of cancer cell lines (i.e., pancreas, breast, prostate, and glioblastoma) and showed a cytotoxic effect at the micromolar range (Rui et al., 2016). In the light of current evidence, pan-SR ligands could represent a new anticancer frontier capable of modulating different molecular cascades.

## Conclusion

In this review, we reported on recent advances in research into SRs, focusing in particular on ER stress and cancer. Both S1R and S2R potentially play a key role in tumor biology as ER stress gatekeepers and are highly expressed in proliferating cancer cells. S1R expression enhances tumor cell aggressiveness by potentiating invasion and angiogenesis, whereas S2R is closely involved in regulating cell proliferation, survival, and invasion. Over the years, numerous compounds have been identified that are capable of binding both receptor types and are endowed with promising anticancer activity. However, further studies are needed to better characterize these enigmatic proteins and unravel their function in cancer biology. Although several potential structures of S1R have been postulated in the last decade, the S1R structure was only elucidated in 2016, thus permitting the design of new chemical entities with a high affinity for this receptor. Conversely, the design of new S2R modulators remains a challenge for researchers because the three-dimensional structure is still unknown. Despite the recent hypothesis that S2R is identical to TMEM97, the 3D structure of the latter has yet to be discovered. Of all the compounds investigated to date, those with an S1R antagonist/S2R agonist profile (i.e., the pan-SR ligands) endowed with excellent anticancer effects, represent a promising strategy to counteract cancer.

In conclusion, we strongly believe that the development of pan-SR drugs is destined to occupy a prominent position in the drug discovery arena and could open up new avenues for the treatment of cancer.

## Author Contributions

AT and SC conceived the research topic, the design of the review, and wrote the manuscript. MR critically contributed to discuss the potential role of SR modulators in cancer therapy. All authors performed the literature review and participated in the drafting and revision of the manuscript, thus making a direct and intellectual contribution to the work.

## Conflict of Interest Statement

The authors declare that the research was conducted in the absence of any commercial or financial relationships that could be construed as a potential conflict of interest.

## Footnotes

1 http://anavex.com/pipeline/

2 https://accuronix.com/approach/

3 http://www.contexttherapeutics.com/our-science/
